# Distorted meta-analytic findings on peer influence: A reanalysis

**DOI:** 10.1016/j.heliyon.2023.e21458

**Published:** 2023-10-24

**Authors:** Kimmo Sorjonen, Gustav Nilsonne, Bo Melin

**Affiliations:** a Department of Clinical Neuroscience, Karolinska Institutet, Stockholm, Sweden; b Department of Psychology, Stockholm University, Stockholm, Sweden; c Meta-Research Innovation Center at Stanford (METRICS), Stanford School of Medicine, Stanford, CA, USA

**Keywords:** Correlated residuals, Cross-lagged panel model, meta-analysis, Peer influence, regression to the mean, Spurious associations

## Abstract

In a recent meta-analysis, Giletta et al. (2021) [1] found a positive effect of peers' behavior at time 1 on target youths' behavior at time 2 while adjusting for target youths’ behavior at time 1 and claimed to have quantified peer influence. However, it is established that controlled cross-lagged effects could be due to correlations with measurement errors and reversion in the direction of the mean rather than due to true decreasing or increasing effects. Here, in a reanalysis of the same meta-analytic data as used by Giletta et al., we found that peer influence, as operationalized by Giletta et al., may have been distorted (i.e. spurious). We do not claim that peer influence does not exist, but it may be hard, maybe not even possible, to prove by analyses of observational data that it does exist. Difficulties to prove causal effects by analyses of observational data is common for all areas of research and not specific for research on peer influence.

## 1 Introduction

According to the influence-compatibility model, the function of peer influence is to increase likeness with peer group affiliates and friends and, thereby, to improve compatibility, increase popularity, and decrease the risk of social exclusion. The need to belong, in turn, may have evolved due to survival and reproductive benefits associated with group membership [1]. In a meta-analysis, Giletta et al. [2] extracted zero-order correlations between target youths' (Y1) and peers' (P1) baseline behavior, as well as target youths’ subsequent behavior (Y2), from the included studies. These behaviors included internalizing (e.g. depression), externalizing (e.g. aggression, substance use), and academic (e.g. homework completion, grades) behaviors. Eq. (1) [3] was employed to calculate the effect of P1 on Y2 while controlling for Y1. A statistically significant meta-analytic effect (β = 0.08) was taken to indicate the occurrence of peer influence [2]. It can be noted that Giletta et al. used zero-order correlations, extracted from the included studies, to estimate controlled cross-lagged effects irrespective of what additional estimates were reported in the included studies.

Eq. 1 $$E\left|\beta_{P1,Y2.Y1}\right|=\frac{r_{P1,Y2}-r_{P1,Y1}r_{Y1,Y2}}{1-r_{P1,Y1}^{2}}$$

However, it is known that effects on subsequent measures of an outcome variable when controlling for prior measures of the outcome may be distorted (i.e. spurious) due to associations with measurement errors and reversion in the direction of the mean [[4], [5], [6], [7]]. As an example, imagine two youths, S and T, with the same measured degree of substance use at baseline, but S has peers with higher measured substance use compared with T. This indicates, with high probability, that S has obtained a lower score than he/she should have, i.e. a negative measurement error, or that T has obtained a higher score than he/she should have, i.e. a positive measurement error. However, as measurement errors tend to reverse in the direction of a mean value of 0 (zero) from one timepoint to the next, we can foresee a more negative, but distorted, change in substance use to a subsequent measurement for T compared with S. Were we to have data from several individuals, we should foresee a positive, but distorted, effect of peers' substance use at time 1 on target youths' substance use at time 2 while controlling for target youths' substance use at time 1. Moreover, as reversion in the direction of the mean does not depend on the direction of time, if the effect is distorted, we should foresee a positive effect of peers' substance use at time 1 on target youths' substance use at time 1 while adjusting for target youths’ substance use at time 2. According to Castro-Schilo and Grimm [4], in nonrandomized studies a difference score model is probably a more adequate alternative than to estimate the effect of a predictor on a measure of the outcome at time 2 while adjusting for a measure of the outcome at time 1.

**Fig. 1:**
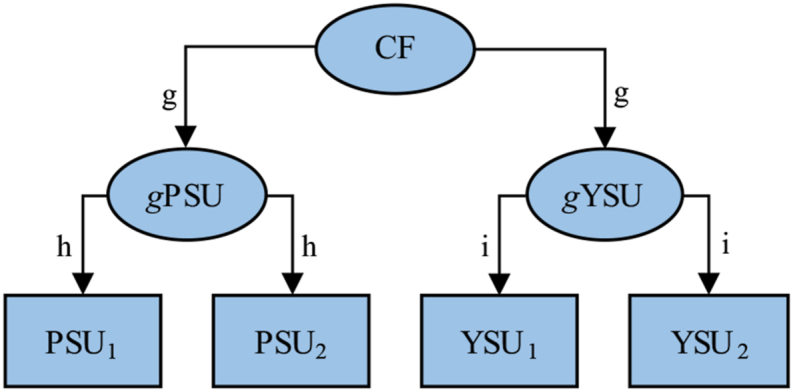
A data generating model where a peer-group common factor (CF, e.g. availability of drugs) affects peers' (*g*PSU) and target youths' (*g*YSU) general substance use which, in turn, affect peers' and target youths' measured substance use on two separate occasions. Although the model does not include any direct effects between peers' and target youths' substance use, adjusted cross-lagged effects, e.g. of PSU_1_ on YSU_2_ when adjusting for YSU_1_, would still be expected to be positive, but spurious, as long as the parameters *g, h,* and *i* are between (but do not include) 0 and 1.

To elaborate, let us assume that data are generated as in Fig. 1, where a peer-group common factor (CF, e.g. availability of drugs) affects peers' general substance use (*g*PSU) and target youths' general substance use (*g*YSU) which, in turn, affect peers' (PSU_1_ and PSU_2_) and target youths' (YSU_1_ and YSU_2_) measured substance use on two occasions. It should be emphasized that in this model there are no direct effects between peers' and target youths’ substance use. In this model, the expected correlation between PSU_1_ and YSU_2_, as well as between PSU_1_ and YSU_1_, equals *h × g × g × i = g*^*2*^*hi*, while the expected correlation between YSU_1_ and YSU_2_ equals *i × i = i*^*2*^. Entering these expected correlations into Eq. (1), we obtain:

Eq. 2 $$E\left|\beta_{PSU1,YSU2.YSU1}\right|=\frac{g^{2}hi-g^{2}hi\times i^{2}}{1-{\left(g^{2}hi\right)}^{2}}=\frac{g^{2}hi\left(1-i^{2}\right)}{1-{\left(g^{2}hi\right)}^{2}}$$

Assuming that all 3 parameters *g, h,* and *i* are above 0 but below 1, both the denominator and the numerator in Eq. (2) will be above 0 but below 1. This means that if data are produced as in Fig. 1, without any direct effect of peers' substance use on target youths' substance use, we should still foresee a positive, but distorted, cross-lagged effect of peers' substance use at time 1 on target youths substance use at time 2 when adjusting for target youths’ substance use at time 1.

**Fig. 2:**
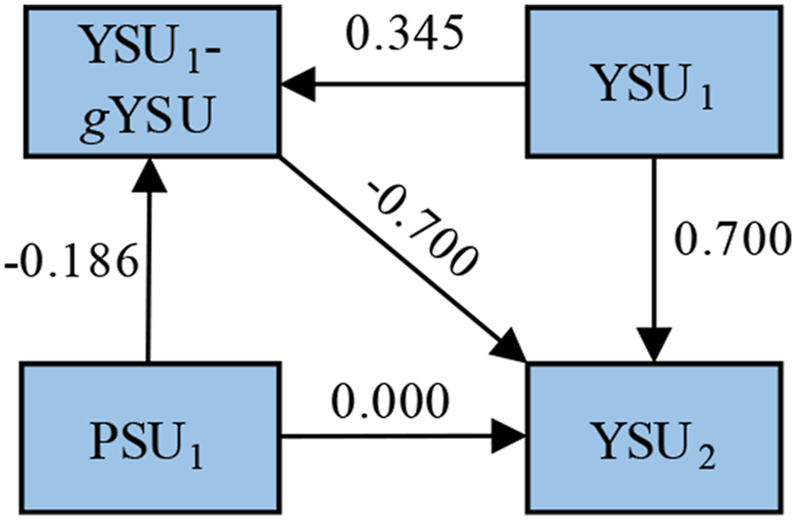
Regression effects between peers' initial substance use (PSU_1_), target youths' initial (YSU_1_) and subsequent (YSU_2_) substance use, and the difference between target youths' initial and general substance use (YSU_1_-*g*YSU, i.e. error in the measurement of target youths' initial substance use). Data were generated as depicted in Fig. 1, with parameters *g, h,* and *i* set to 0.7 (*N* = 100,000).

To exemplify, we conducted a simulation (*N* = 100,000) where data was produced as in Fig. 1, with no direct effects between peers' and target youths' substance use, with all three parameters *g, h,* and *i* set to 0.7. As expected (given Equation (2)), the total effect of peers' initial substance use (PSU_1_) on target youths' subsequent substance use (YSU_2_) when adjusting for target youths' initial substance use (YSU_1_) was equal to 0.130 (in Fig. 2 this corresponds to −0.186 × −0.700). However, in accordance with the discussion above, when adjusting for YSU_1_, PSU_1_ had a negative association with the YSU_1_ – *g*YSU (target youths’ general substance use) difference, i.e. error in the measurement of YSU_1_ (β = −0.186). When adjusting for the YSU_1_ – *g*YSU difference in addition to YSU_1_, PSU_1_ no longer had any effect on YSU_2_ (β = 0.000). This means that when adjusting for YSU_1_, the effect of PSU_1_ on YSU_2_ was solely due to a correlation between PSU_1_ and error in the measurement of YSU_1_ combined with an inclination for measurement errors to reverse in the direction of a mean value of zero from one timepoint to the next, i.e. the effect was distorted.

The goal of this reanalysis was to assess if the peer influence claimed by Giletta et al. [2], based on a meta-analytic adjusted regression effect, could have been distorted due to correlation with measurement errors and reversion in the direction of the mean.

## 2 Method

See Giletta et al. [2] for more comprehensive information on choice of studies, tests of publication bias, and description of the included studies. In short, Giletta et al. extracted zero-order correlations between peers' initial behavior and target youths' initial and subsequent behavior from 60 longitudinal studies (total *N* = 47,423, mean age at baseline = 12.5 years, *Mdn* = 52 % girls) and used Eq. (1) to estimate adjusted regression effects. A list of included studies is available at https://osf.io/q7mwa. As Giletta et al. received almost identical results if limiting estimations to consecutive waves of measurement (155 effect sizes), compared with estimations using all pairwise assessments (233 effect sizes), we limited our analyses to consecutive waves of measurement. Moreover, compared with Giletta et al., we excluded two studies from our analyses (with a total of three effect sizes) that did not appear to present zero-order correlations. We did this in order to be consistent across the three estimations of regression effects. Hence, our estimations were based on 152 effect sizes from *K* = 58 independent studies with a total sample size of *N* = 46,945. The exclusion of three effect sizes had hardly any effect on the meta-analytically estimated effect of peers' behavior at time 1 on target youths' behavior at time 2 while adjusting for target youths’ behavior at time 1 (the effect remained at β = 0.08).

### 2.1 Statistical analyses

Similarly as Giletta et al. [2], we employed Eq. (1) to calculate the effect of peers' behavior at time 1 on target youths' behavior at time 2 while adjusting for target youths' behavior at time 1. Additionally, we employed Eq. (1) to calculate the effect of peer's behavior at time 1 on target youths' behavior at time 1 while adjusting for target youths' behavior at time 2. With a genuine peer influence, this latter effect was expected to be negative, which would mean that among target youths with the same subsequent degree of the behavior, those with peers with a higher initial degree of the behavior tended to have a lower initial degree of the behavior and had, consequently, experienced a more positive (quantitatively) change in the behavior from time 1 to time 2 compared with target youths with the same degree of the behavior at time 2 but with peers with a lower degree of the behavior at time 1. As an example, imagine two youths, S and T, with the same subsequent degree of substance use (e.g. the mean standardized value 0), but S has peers with higher initial degree of substance use compared with T. With a genuine peer influence we would foresee a higher degree of substance use at time 1 for T (e.g. 0.5) than for S (e.g. −0.5) as this would suggest a more negative change in substance use from time 1 to time 2 for T (0–0.5 = −0.5) than for S (0 – (−0.5) = 0.5). Contrarily, if the estimated peer influence was distorted due to associations with measurement errors and reversion in the direction of the mean, the effect was expected to be positive.

Moreover, we used Eq. (3) [8] to calculate the effect of peers' behavior at time 1 on target youths’ time 2 – time 1 behavior difference. A hypothesis of genuine peer influence forecasted this effect to be positive. Contrarily, a hypothesis of a distorted effect forecasted either an effect not far from to zero (if contemporaneous, *r*_*P1,Y1*_, and cross-lagged, *r*_*P1,Y2*_, correlations were approximately equal in size) or a negative effect (if the contemporaneous correlation was more positive than the cross-lagged correlation).

Equation 3 $$E\left|\beta_{P1,Y2-Y1}\right|=\frac{r_{P1,Y2}-r_{P1,Y1}}{\sqrt{2\left(1-r_{Y1,Y2}\right)}}$$

We performed a multilevel meta-analysis with random effects for all three regression effects described above, as well as for each of the three zero-order correlations. Independent effect sizes were used to estimate a meta-analytic effect (random). Analyses were performed on Fisher's z-converted regression effects (standardized), but for presentations these were reversed back to non-converted effects. Analyses were conducted with R 4.3.1 statistical software [9] using the metafor package [10]. Data and analytic script are accessible at the Open Science Framework at https://osf.io/nkpc6/.

## 3 Results

**Fig. 3:**
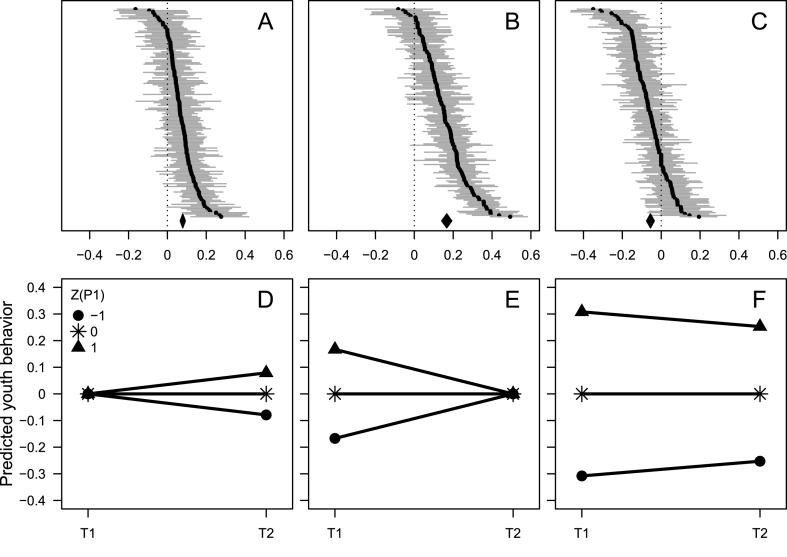
(A–C) Caterpillar plots of 152 ordered effects (with 95 % CI) of peers' initial behavior on target youths' subsequent (A) or initial (B) behavior, or on the subsequent behavior – initial behavior difference (C), while adjusting for target youths' initial (A) or subsequent (B) behavior, or with no adjustment (C). The diamond indicates the meta-analytic effect (with 95 % CI); (D–F) Predicted initial and subsequent target youths' behavior separately for those whose peers exhibited high (Z (P1) = 1), average, or low (Z (P1) = −1) degree of the behavior in question at the initial measurement, when conditioning on average initial (D) or subsequent (E) target youths' behavior, or without conditioning (F). Note: Target youths' predicted subsequent behavior (Y2) was a function of target youths' initial behavior (Y1) and peers' initial behavior (P1) according to the equation: E|Y2| = β_1_ × Y1 + 0.079 × P1 (see Table 1). Consequently, if conditioning on average initial target youths' behavior (Y1 = 0), subsequent target youths' behavior was predicted to be β_1_ × 0 + 0.079 × -1 = −0.079, β_1_ × 0 + 0.079 × 0 = 0, and β_1_ × 0 + 0.079 × 1 = 0.079 for those with peers who exhibited the behavior to a low (Z (P1) = −1), average (Z (P1) = 0), and a high (Z (P1) = 1) degree, respectively. This is illustrated in panel D. Target youths' predicted initial behavior (Y1) was a function of target youths' subsequent behavior (Y2) and peers' initial behavior (P1) according to the equation: E|Y1| = β_1_ × Y2 + 0.167 × P1 (see Table 1). Consequently, if conditioning on average subsequent target youths' behavior (Y2 = 0), initial target youths' behavior was predicted to be β_1_ × 0 + 0.167 × -1 = −0.167, β_1_ × 0 + 0.167 × 0 = 0, and β_1_ × 0 + 0.167 × 1 = 0.167 for those with peers who exhibited the behavior to a low (Z (P1) = −1), average (Z (P1) = 0), and a high (Z (P1) = 1) degree, respectively. This is illustrated in panel E. If not conditioning, target youths' predicted initial behavior (Y1) was a function of peers' initial behavior (P1) according to the equation: E|Y1| = 0.308 × P1, and predicted change in Y was a function of P1 according to the equation: E|ΔY| = −0.055 × P1 (see Table 1). Consequently, Y1 was predicted to be 0.308 × -1 = −0.308, 0.308 × 0 = 0, and 0.308 × 1 = 0.308 for those with peers who exhibited the behavior to a low (Z (P1) = −1), average (Z (P1) = 0), and a high (Z (P1) = 1) degree, respectively. Change in Y was predicted to be −0.055 × -1 = 0.055, −0.055 × 0 = 0, and −0.055 × 1 = −0.055 for those with peers who exhibited the behavior to a low, average, and a high degree, respectively. Consequently, target youths' subsequent behavior was predicted to be −0.308 + 0.055 = −0.253, 0 + 0 = 0, and 0.308–0.055 = 0.253 for those with peers who exhibited the behavior to a low, average, and a high degree, respectively. This is illustrated in panel F.

Meta-analytically estimated zero-order correlations as well as controlled regression effects between peers' initial behavior and target youths' initial and subsequent behavior, or difference between these two, are presented in Table 1. Estimated associations, aggregated across the 58 included studies, exhibited statistically non-significant and mostly low heterogeneity, as estimated by *I*^*2*^, which corresponds to proportion of variation across effects that can be attributed to heterogeneity rather than random variation, and Cochran's *Q*. The zero-order correlations (row 1–3) were substantial. According to Eq. (1), a strong autocorrelation between target youths' behavior at time 1 and time 2 had a decreasing impact on the cross-lagged effect of peers' behavior at time 1 on target youths' behavior at time 2 while controlling for target youths' behavior at time 1. The effect of peers' behavior at time 1 on target youths' behavior at time 2 while adjusting for target youths' behavior at time 1 was weak but positive and statistically significant (row 4 in Table 1 and panel A in Fig. 3). This means that among target youths who initially exhibited the behavior to the same degree, those with peers who initially exhibited the behavior to a higher degree tended to experience a quantitatively more positive change in the behavior between time 1 and time 2 compared with target youths with peers who exhibited the behavior to a lower degree (panel D in Fig. 3).

**Table 1:** Meta-analytic zero-order correlations and adjusted regression effects between peers' and target youths’ initial and subsequent behavior.

Parameter	Estimate (95 % CI)	*Q* (df)	*I*^2^ (95 % CI)
1. r (P1,Y1)	0.308 (0.272; 0.343)	57 (57)	0.0 (0; 33.7)
2. r (Y1,Y2)	0.582 (0.549; 0.614)	54 (57)	0.0 (0; 29.5)
3. r (P1,Y2)	0.254 (0.225; 0.283)	57 (57)	0.8 (0; 35.4)
4. β(P1,Y2.Y1)^1^	0.079 (0.066; 0.092)	56 (57)	0.0 (0; 35.5)
5. β(P1,Y1.Y2)^1^	0.167 (0.141; 0.193)	58 (57)	1.7 (0; 35.6)
6. β(P1,Y2–Y1)^1^	−0.055 (−0.075; −0.035)	58 (57)	1.4 (0; 36.1)

Note: *Q* = Cochran's Q; *I*^*2*^ = percentage of variation due to heterogeneity; P1, Y1, and Y2 = peers' and target youths' initial and subsequent behavior, respectively; ^1^ the variables are given in the order predictor, outcome, and covariate.

However, contrary to a hypothesis of genuine peer influence, and in accordance with a hypothesis of a distorted effect due to associations with measurement errors and reversion in the direction of the mean, the effect of peers' behavior at time 1 on target youths' behavior at time 1 while adjusting for target youths’ behavior at time 2 was also positive and statistically significant (row 5 in Table 1 and panel B in Fig. 3). This means that among target youths who subsequently exhibited the behavior to the same degree, those with peers who initially exhibited the behavior to a higher degree tended to have experienced a quantitatively more negative change in the behavior from time 1 to time 2 compared with target youths with peers who exhibited the behavior to a lower degree (panel E in Fig. 3).

Also contrary to a hypothesis of genuine peer influence, if not conditioning on target youths' initial behavior, the effect of peers' behavior at time 1 on target youths’ time 2 – time 1 behavior difference was, although weak, negative and statistically significant (row 6 in Table 1 and panel C in Fig. 3). This means that target youths with peers with a high degree of the behavior in question tended themselves to exhibit the behavior to a high degree initially, but to experience a quantitatively more negative change in the behavior between time 1 and time 2 compared with target youths with peers with a lower degree of the behavior (panel F in Fig. 3).

## 4 Discussion

The findings of the present reanalysis suggested that the meta-analytically estimated peer influence by Giletta et al. [2] may have been distorted. Although peers' behavior at time 1 had a significant (statistically) positive effect on target youths' behavior at time 2 even when controlling for target youths' behavior at time 1, the same was true when using target youths' initial behavior as the outcome and subsequent behavior as the covariate. Furthermore, when not adjusting for the target youths' initial behavior, the peers' initial behavior had a negative effect on target youths’ subsequent – initial behavior difference.

Hence, the present analyses indicated, simultaneously, both an increasing and a decreasing influence of the behavior of peers on the behavior of target youths. This contradiction is a strong indication that the findings may have been distorted, probably due to associations with measurement errors and reversion in the direction of the mean. As an example, imagine two youths, S and T, with the same measured degree of substance use at baseline, but S has peers with higher measured substance use compared with T. This indicates, with high probability, that S has obtained a lower score than he/she should have, i.e. a negative measurement error, or that T has obtained a higher score than he/she should have, i.e. a positive measurement error. However, as measurement errors tend to reverse in the direction of a mean value of 0 (zero) from one timepoint to the next, we can foresee a more negative, but distorted, change in substance use to a subsequent measurement for T compared with S.

It should be emphasized that we do not claim that influence from peers does not exist. However, as generally, a causal effect is difficult to prove with correlational data. We believe that this limitation should be acknowledged by researchers. Cross-lagged effects while controlling for a baseline score on the outcome variable do often not indicate anything except a cross-sectional correlations in combination with measurement errors. Researchers should be aware of this limitation of cross-lagged effects so they do not over-interpret findings, something that may be the case in Giletta et al. [2]. For research on peer influence, stochastic actor-oriented models have been developed to discriminate between genuine peer influence and similarity due to social selection, i.e. that adolescents who are more alike tend to befriend to a higher degree than adolescents who are less alike [[11], [12], [13], [14]]. Findings from stochastic actor-oriented models are, we assume, expected to be less susceptible to distorted findings due to associations with measurement errors and reversion in the direction of the mean compared with findings from cross-lagged panel analyses. However, we estimated peer influence with a stochastic actor-oriented model in data generated similarly as in Fig. 1, with no direct effects between peers' and target youths’ behavior, and found a statistically significant increasing effect of the behavior of peers on the behavior of target youths (see supplementary documents SAOM_Script and SAOM_Results at https://osf.io/nkpc6/). Although a preliminary finding, this suggests that stochastic actor-oriented models may be susceptible to distorted findings in a similar way as cross-lagged panel models.

Researchers often seem to assume that by using the right statistical method, they can unearth true causal effects in correlational (i.e. non-experimental) data. We believe this assumption to be overly optimistic. As stated above, trying to prove causal effects with observational data is probably a futile endeavor in most cases. Although this might sound pessimistic, we do not believe that false hopes would serve the research community well. We should be aware of limitations inherent in our work. What researchers can do, however, is to analyze, as we have done here, models that make dissimilar forecasts if associations are truly increasing or decreasing or distorted. If results from different models converge, conclusions of causality may be seen as corroborated (although never proven with absolute certainty). If, on the other hand and as in the present study, findings diverge, associations may be distorted and conclusions of causality would probably be premature.

It should also be noted that we do not wish to make claims about nor criticize all research on peer influence. There are probably many findings in this research area that are not due to regression to the mean. For example, adolescents appear to be influenced more by popular than by less popular peers [[15], [16], [17], [18]], a moderation that would be difficult to explain as a purely distorted consequence of reversion in the direction of the mean. The present study should, rather, be seen as a critique of studies, e.g. Giletta et al. [2], with overinterpretations of findings from cross-lagged panel analysis.

We have previously pointed out other examples of longitudinal analyses with spurious results when adjusting for baseline/earlier levels of the outcome and recommended that researchers can make use of estimations with inverted direction of time, as in the present study, to help assess the risk of distorted findings [[19], [20], [21], [22], [23], [24], [25], [26], [27]]. The continuing flow of published papers using baseline adjustment in longitudinal analyses and risking distorted results as a consequence suggests that the problems associated with doing so, although well described, are still not well known throughout the academic community. The present paper adds to the body of knowledge suggesting that greater awareness of this problem among researchers is called for.

### 4.1 Limitations

This reanalysis suffers from some similar limitations as the challenged meta-analysis by Giletta et al. [2]. For example, a vast majority of the included studies (92 %) were conducted in Western countries and with mainly White participants. Consequently, we cannot conclude with certainty that the key finding of the present reanalysis, namely that peer influence in correlational data appears to be distorted, applies to other populations.

It is likely that not all included studies used optimal measures of peers' and target youths' behavior. Moreover, possibly moderating variables such as time from one measurement to the next, proportion of female and male participants, the participants’ age, etc., were not considered in the present reanalysis. However, it should be emphasized that such factors did not vary between the analyzed models. Therefore, they cannot account for why the models suggested, simultaneously and paradoxically, both an increasing and a decreasing effect of the behavior of peers on the behavior of target youths.

## 5 Conclusions

Based on a meta-analytic regression effect of peers' behavior at time 1 on target youths' behavior at time 2 while adjusting for target youths’ behavior at time 1, Giletta et al. [2] claimed to have quantified peer influence. However, our reanalysis of the same meta-analytic data suggested, contrarily, that the observed peer influence may have been distorted due to associations with measurement errors and reversion in the direction of the mean.

## Data availability statement

Data and analytic script are available at the Open Science Framework at https://osf.io/nkpc6/.

## Ethics declaration

Review and/or approval by an ethics committee was not needed for this study because it only included analyses of secondary and publicly available data. Informed consent was not required for this study because it only included analyses of secondary and publicly available data.

## CRediT authorship contribution statement

**Kimmo Sorjonen:** Conceptualization, Data curation, Formal analysis, Investigation, Methodology, Project administration, Software, Visualization, Writing – original draft, Writing – review & editing. **Gustav Nilsonne:** Conceptualization, Investigation, Methodology, Supervision, Validation, Writing – review & editing. **Bo Melin:** Conceptualization, Investigation, Resources, Supervision, Writing – review & editing.

## Declaration of competing interest

The authors declare that they have no known competing financial interests or personal relationships that could have appeared to influence the work reported in this paper.
